# Supplementary material for: Influence of adiposity and sex on SARS-CoV-2 antibody response in vaccinated university students: A cross-sectional ESFUERSO study
Source: PLOS Glob Public Health. 2024 Jul 26;4(7):e0002686. doi: 10.1371/journal.pgph.0002686 (PMC11280215; doi:10.1371/journal.pgph.0002686)
Supplement: Attachment — Submitted filename: Peer review comments from Mateus_Art5.docx [file pgph.0002686.s003.docx]

**Peer review comments from Mateus**

**Title:**

Adiposity and Sex Influence on SARS-CoV-2 Antibody Response in University

Students. An ESFUERSO cross-sectional study.

**Abstract**

Line 66: “… this trend was …” => which trend?

**Introduction**

Line 76: Insert references after the word conducted.

Line 80: Why reference comes after a dot (.)? => .(1) should read (1). Please correct this in the whole document.

Line 87: “BMI > 40 kg/m2” should read “BMI > 40 kg/m^2^”.

Line 90: If ESFUERSO is an abbreviation, can it be explained in full here?

Line 88: Insert reference after the word responses.

Lines 91-92: “… which recruited first-year college students living in Reynosa prior the

COVID-19 pandemic in 2018”. => this sentence should be part of methods section, not introduction.

Lines 92-99: “This group of students reported … psychological stress”. This paragraph looks like results rather than introduction. Can this be clarified?

Lines 99-100: “Vaccination 99 efforts began in 2021, employing novel mRNA vaccine used in veterinary science for three decades”. => Did authors do vaccination as part of this study (method)? If not, then this sentence needs reference if it comes from the literature.

Lines 102-103: “The aim of this study was …” => start this as a new paragraph.

Lines 105-106: “The study provides insights in the immune response and the potential implications in the context of COVID-19”. => This sentence should come later in the results or discussion.

**Comment for the introduction**: This section of the manuscript is too short, less than one page is not sufficient. There is no clear rational of the study described here. Authors need to screen further literature to make this section rich and explain why this study is important and/or needed.

**Methods**

Line 111: Better to avoid confusion: is the ESFUERSO study the one that is being published in this manuscript or different? How sample size was calculated? 500? 116? 108? Better to stick on sample size for your manuscript and explain how you got this figure. Did the authors check the power for this sample size to be representative of the studied population? A subset of 116, was it for what (survey, blood collection or both)?

Line 110: Based on the content of this paragraph, this title should be: Study sample, design, and data collection. Please describe the study design for your research (it seems to be a quantitative clinical study and comparative, I guess). Did the authors respect GCP procedures? Were there SOP and training of study staff done? What was done for the safety of study participants and staff? Explain in details how survey was conducted and how participants were selected (sampling method). Explain in details what anthropometric measurements were done. What did authors do with blood samples collected? What lab facility was used for analysis, for what kind of results?

Line 127: “… the Coping Inventory for Stressful Situations (SISCO),…” => is it CISCO or CISS? Please check. It seems that the system used was CISS, not CISCO. Is there any relationship between stress and adiposity? It is not seen in the background.

Lines: 130-142: Why this section is under Measures? It is better to have another sub-heading named “Data collection” and put this information there. The Measures could contain only information about weight and height and how it was done. Were the materials used calibrated and quality checked? If the survey was administered electronically, what if participants needed clarifications for some questions before responding? What was put in place to solve that and avoid bias in the responses provided?

Lines 134-136: **“Blood samples were obtained after an overnight fasting period for the measurement of serum concentration of Ig-G anti-RBD by indirect ELISA”** => What was the real time set for blood samples collection to avoid participants being kept fasting for long time? Was there payment for time and transport compensation? As the survey was online, how participants who gave blood get in contact with study staff to provide their blood? Was there a dedicated study site where they could go or staff could move to their home to collect blood?

Lines 144-148: Was the consent voluntary or paid? Was there any study information sheet given to participants? Did they get explained the study procedure before accepting to take part of it? Were they given opportunity to withdraw from the study any time when they feel to do so?

Lines 151-152: “Descriptive statistics with percentage for counts variables, mean and standard deviation for continuous variables”. => This is not a sentence, please correct grammar issue here.

Line 152: CISCO or CISS?

Line 158: “… lineal models …” or “linear models”?

If you are trying to link adiposity and immunogenicity, how the CISS components fit into the analysis here? Are you comparing the level of immunity of people who are affected by stress to those with adiposity? Please clarify. CISS components can be cofounding factors, how do you deal with that? There is possibility of having 3 groups of participants to compare: those with stress only, those with adiposity only, and those with both stress and adiposity. Please specify what you are trying to do for this study. It is still not clear.

Also specify what are the inclusion/exclusion criteria for participants. Did you include people who are only vaccinated (fully, partially, or not vaccinated)? How did you check the vaccination status? Or did you vaccinate them yourself as a prospective study? How did these criteria apply for survey and blood collection?

What kind of analysis did you perform (univariate, bivariate or both)?

Line 161: “… with 114 students with all complete data”. => What is the real sample size for this study (500, 116, 108, 114)?

Add a title of “Data presentation” under which you explain how your data are presented in this manuscript: graphs (bar chart, trend lines, pie chart, …), table, text, etc.

**Results**

Line 165: “… 108 students …” => Be consistent, what is the real sample size for this study?

Lines 165-176: We do not see the data here. There is interpretation of data that are not presented here. Please present your data before making any interpretation or comment.

Line 176: What do authors mean by “positive anti-RBD”?

Line 177: In the table “BMI (kg/m^2)” should read “BMI (Kg/m^2^)

Line 177: Data in table 1 are confusing to me. Please explain what is in brackets, dot (.) for some and comma (,) for others.

The way table 1 is presented, it looks like sex is the dependent variables that you are trying to link to all other variables in the first column. It could be better to have sex in the same column with the other variable (column 1) and put data about immunogenicity at the place of sex. Did you have sub categories for the independent variables (e.g. for age: young, medium adult and older)? Was the immunity the same for all of them?

Lines 184-196: This seems to be a bivariate analysis linking sex and adiposity. This should be clearly specified in the method. Why comparison between males and females only? You should do the same for other variables too. You should also compare fat and non-fat people, people with hypertension and those with normal blood pression, older and young, those with metabolic risk factors with those without risk factors, etc.

Line 188: Ab-RBD: What this abbreviation means? Better to be consistent in the whole document.

Lines 194-196: This is an interpretation of data that are not presented in the manuscript. Can you present a figure here before making interpretation?

You have only 2 figures, all other data are not presented but have interpretation and comment.

Were males and females receiving the same type of vaccine? This should be clarified. If they received different type of vaccine, it may be difficult to compare these findings!

**Discussions**

Lines 214-215: “Stress scores do not appear to have significant effects” => I did not see data in your results showing this.

Lines 216-219: The introductory sentence in this paragraph gives link between sex and immune response but the example given link sex and adiposity. It is better to give the right example here.

Line 229: Insert reference after the word “index (BMI)”.

Lines 230-232: “This study highlighted the heightened vulnerability of individuals with underlying health conditions or obesity to SARS-CoV-2 infection” => I did not see data in the analysis supporting this statement.

Line 235: “Other populations have reported similar findings …” => what authors mean by other population? Does it mean study conducted out of Mexico? It should be clear.

Line 242: “… this outcomes” should read “these outcomes”.

**Note:** I realise that there is no conclusion in this manuscript. Please add this section. Lines 243-248 should be part of the conclusion. Authors may add other important information such open window for new research. It is important to have research looking at COVID-19 vaccine protection level in a population with other weight for example.

**Conclusion**

To be added, see comment above.

**General comments:**

This is a very good study that add value to the literature. However, it need improved by considering the above comments before moving to publication.
