# Supplementary material for: Influence of adiposity and sex on SARS-CoV-2 antibody response in vaccinated university students: A cross-sectional ESFUERSO study
Source: PLOS Glob Public Health. 2024 Jul 26;4(7):e0002686. doi: 10.1371/journal.pgph.0002686 (PMC11280215; doi:10.1371/journal.pgph.0002686)
Supplement: Attachment — Submitted filename: PLOS GPH Re-review for Perales-Torres et al. April 2024.docx [file pgph.0002686.s005.docx]

Re-Review for Perales-Torres et al.

**Introduction**

The introduction is improved. However, the whole paper would benefit from a conceptual diagram (even a simple one), as well as an objective statement, that connects some of the concepts in the introduction (that you added this round) to your study methods. In your response to reviewer 5 you mentioned that you kept the introduction short to keep things concise: I don’t think it’s about literal length per se, so much as you still have not motivated the study yet in this round of revisions. Right now, thinking from the perspective of a naïve reader, it’s not clear why you selected the methods you did because the research question and how you intend to answer that research question are still not clear. E.g. It is not clear to me why you evaluate *these seven models*? Why does a reader want to look at stress as measured by SISCO here? Why should a reader want to know about the effects of your independent variables on RBD? That’s why it’s not clear from the text whether this paper is exploratory analysis—something I mentioned last round—while your response to reviewers makes it clear that you do have specific hypotheses (and which you begin to hint at this round in the introduction). If you have hypotheses, *make them explicit* to the reader in an objectives statement. A conceptual diagram, with the components explained and referenced in the text of the Introduction, will also help you make explicit what exactly you’re operationalizing using your measures and why. Otherwise, there is not enough information for reviewers or readers to assess the *internal validity* of your study. Your response to reviewers (both to me and to reviewer 5) explains this relatively well, that level of explicit explanation should be in the paper itself.

89-90: needs a reference (unless it’s meant to refer to citation (7), in which case the sentence should be edited for clarity).

99: needs something at the beginning of the sentence to make clear that you’re referring to widespread vaccination efforts, rather than an activity that your study carried out. E.g. “Country-wide vaccination efforts…”

104: needs a citation for the protective epitope. And needs more background explanation + references here to explain why it’s important to study RBD and IgG and their ratio.

**Methods**

Overall, methods and its relationship to the introduction is somewhat improved since last draft, enabling some evaluation of the statistical methods.

The Study Sample section is much improved especially regarding sample size and how that number was arrived at.

There is still no necessary detail on the method by which many key anthropometric data were collected. E.g. was body fat percentage by caliper? (It seems maybe bioimpedance? according to Figures 1 and 2, but should be in the text). The authors need to go through the paper with the perspective of a reader trying to understand the methods used.

There is still not enough information in the statistical analysis section on your regression analyses. You need to describe each of the 7 models (even if just briefly) more than merely mentioning that there are 7 in 175. You also need to state what you consider your threshold for statistical significance.

125-127: The added explanations of SISCO and what you meant by metabolic risk score are very helpful to the reader.

139-141: Is effective neutralization >1 or <1? From context of your use of “positive anti-RBD”, I guess that it’s >1, but you need to make this explicit for the reader.

143-148: In terms of logical flow of section order, the ethics statement may fit better if it came right after the “study sample” section.

154-158: This is more a set of data measures, rather than statistical methods, and should be moved to the measures section.

164-174: it is not necessary to go into this great of detail about VIF in the methods section. The much better explanation in 200-202 is all that is needed for a reader unfamiliar with VIF to be able to interpret VIF.

**Results**

196: Table 1: Your measures of positive anti-RBD and metabolic risk score should be in Table 1 as well.

196: Table 1: Food factors show up in Table 2 (as it should), and should show up here in Table 1 as well, as stated in my last round of suggestions. Again, this goes back to the importance of motivating the study well in the Introduction and making explicit your hypotheses. Why was food something you considered, even if it ended up not significant?

Table 2: I appreciate the addition of Table 2, in which you appear to report all results, and not just the significant ones. Table 2 makes the paper much more cohesive.

Table 2: 95% CI would be much more interpretable to a reader than standard errors.

200-202: this is a good added explanation of how a reader can interpret your VIF.

**Discussion**

Overall, the findings in the discussion section seem consistent with the newly reported results in table 2.

238-263: as mentioned in the last round, these sources should be part of your introduction to help motivate the study, since they would presumably inform the hypotheses that you’re testing.

259: meta-analysis should not be capitalized.

265: your study is on sex differences, not gender differences.
