# Supplementary material for: Influence of adiposity and sex on SARS-CoV-2 antibody response in vaccinated university students: A cross-sectional ESFUERSO study
Source: PLOS Glob Public Health. 2024 Jul 26;4(7):e0002686. doi: 10.1371/journal.pgph.0002686 (PMC11280215; doi:10.1371/journal.pgph.0002686)
Supplement: Attachment — Submitted filename: 240613_Response to Reviewers.docx [file pgph.0002686.s006.docx]

Edinburg, Texas June 13, 2024.

Re-Review for Perales-Torres et al.

*We appreciate the time and effort you dedicated to reviewing our paper, and we value the comments we received as they significantly enhance the manuscript*.

**1. Introduction**

**1.1. The introduction is improved**. However, the whole paper would benefit from a conceptual diagram (even a simple one), as well as an objective statement, that connects some of the concepts in the introduction (that you added this round) to your study methods. In your response to reviewer 5 you mentioned that you kept the introduction short to keep things concise: I don’t think it’s about literal length per se, so much as you still have not motivated the study yet in this round of revisions. Right now, thinking from the perspective of a naïve reader, it’s not clear why you selected the methods you did because the research question and how you intend to answer that research question are still not clear. E.g. It is not clear to me why you evaluate *these seven models*?

**Answer***. Thank you for this valuable commentary. We have revised the introduction to clarify the rationale for our study and the selection of variables for our analysis better. Current literature indicates that stress can alter the immune response to vaccines and that there have been reports of weight gain during the pandemic due to stress and increased food intake. Consequently, we investigated the vaccine response, considering various psychological, behavioral, and biological factors that might influence it. Our models were designed to address these research questions, with sex being a consistent factor across all models tested. Additionally, we have included a conceptual diagram illustrating the initial hypothesized relationships and the outcomes to provide a clearer visual representation of our study’s framework.*

**1.2. Why does a reader want to look at stress** as measured by SISCO here? Why should a reader want to know about the effects of your independent variables on RBD? That’s why it’s not clear from the text whether this paper is exploratory analysis—something I mentioned last round—while your response to reviewers makes it clear that you do have specific hypotheses (and which you begin to hint at this round in the introduction). If you have hypotheses, *make them explicit* to the reader in an objectives statement. A conceptual diagram, with the components explained and referenced in the text of the Introduction, will also help you make explicit what exactly you’re operationalizing using your measures and why.

**Answer**. *Thank you for your insightful comment. Our study follows the guidelines set by NIH and CONAhCyT, which require sex analysis, a standard practice for our research group over the years. We understand that this focus may be less familiar to other readers. Your suggestion for a conceptual diagram was very helpful, and we have created one to elucidate these issues.*

*The SISCO questionnaire was chosen because it explicitly measures stress during the pandemic, allowing us to capture the unique psychological stressors of this period. We have also added relevant literature to the introduction to highlight the impact of psychological variables on the immune response, providing a stronger rationale for our study.*

*To address our specific hypotheses, we have included a conceptual diagram that clarifies what we are operationalizing with our measures to understand their effects on RBD.*

**1.3. Otherwise, there is not enough information for reviewers** or readers to assess the *internal validity* of your study. Your response to reviewers (both to me and to reviewer 5) explains this relatively well, that level of explicit explanation should be in the paper itself.

**Answer**. *Thank you for your feedback. We appreciate your concern regarding the assessment of internal validity. In the manuscript, lines 120 to 132 detail the internal validity of the questionnaires used in our study. Additionally, we have referenced the ESFUERSO study, which offers comprehensive information on the validity and reliability of these measures. To ensure this information is easily accessible to reviewers and readers, we will make these details more explicit in the text, highlighting the steps taken to confirm the internal validity of our study's instruments.*

**1.4. 89-90: needs a reference** (unless it’s meant to refer to citation (7), in which case the sentence should be edited for clarity).

**Answer**. *Thank you for this observation. We moved the reference at the end of the second sentence (line 90) to increase the clarity of the statement*.

**1.5. 99: needs something at the beginning of the sentence** to make clear that you’re referring to widespread vaccination efforts, rather than an activity that your study carried out. E.g. “Country-wide vaccination efforts…”

**Answer**. *Thank you for the suggestion. We have rephrased the sentence to provide more context on the research done in RNA vaccination: “The development of mRNA vaccines has been a long journey, beginning with cell cultures and laboratory animal testing, followed by industry investment and veterinary applications, ultimately leading to human vaccination in 2020. These efforts have been ongoing for three decades.”(sic)*

**1.6. 104: needs a citation** for the protective epitope. And needs more background explanation + references here to explain why it’s important to study RBD and IgG and their ratio.

**Answer**. *We added two more references on this issue. Thank you for the observation*.

**2. Methods**

Overall, methods and its relationship to the introduction is somewhat improved since last draft, enabling some evaluation of the statistical methods. The Study Sample section is much improved especially regarding sample size and how that number was arrived at.

**Answer***. We are glad you agreed with the changes we made*.

**2.1. There is still no necessary detail** on the method by which many key anthropometric data were collected. E.g. was body fat percentage by caliper? (It seems maybe bioimpedance? according to Figures 1 and 2, but should be in the text). The authors need to go through the paper with the perspective of a reader trying to understand the methods used.

**Answer**. *We rewrote the paragraph. Thank you for this important observation*.

**2.2. There is still not enough information** in the statistical analysis section on your regression analyses. You need to describe each of the 7 models (even if just briefly) more than merely mentioning that there are 7 in 175. You also need to state what you consider your threshold for statistical significance.

**Answer***. We decided to maintain only five models (instead of the initial seven) to make Table 2 clearer. We agreed with your suggestion to use 95% CI (below in comment 3.4), but the table looks full of numbers. The new Table 2 looks good with two fewer models*.

**2.3. 125-127: The added explanations of SISCO** and what you meant by metabolic risk score are very helpful to the reader.

**Answer**. *We clarified that SISCO was proposed during the COVID-19 pandemic to evaluate the stress of patients.*

**2.4. 139-141: Is effective neutralization >1 or <1?** From context of your use of “positive anti-RBD”, I guess that it’s >1, but you need to make this explicit for the reader.

**Answer**. *Thanks for the observation; this concept is stated in lines 140-142*.

**2.5. 143-148: In terms of logical flow** of section order, the ethics statement may fit better if it came right after the “study sample” section.

**Answer**. *We modified the order of the sections*.

**2.6. 154-158: This is more a set of data measures**, rather than statistical methods, and should be moved to the measures section.

**Answer**. *We modified the paragraph and moved it to the Methods Section*.

**2.7. 164-174: it is not necessary to go into this great of detail** about VIF in the methods section. The much better explanation in 200-202 is all that is needed for a reader unfamiliar with VIF to be able to interpret VIF.

**Answer**. *Thanks for your recommendation. We made changes on the technical information*.

**3. Results**

**3.1. 196: Table 1: Your measures of positive anti-RBD** and metabolic risk score should be in Table 1 as well.

**Answer**. *Thank you for your feedback. In response to your suggestion, we have updated Table 1 to include the prevalence of positive anti-RBD by sex. Additionally, we have decided to eliminate the metabolic risk score from the paper for clarity and focus (It did not added new information in this paper).*

**3.2. 196: Table 1: Food factors show up** in Table 2 (as it should), and should show up here in Table 1 as well, as stated in my last round of suggestions. Again, this goes back to the importance of motivating the study well in the Introduction and making explicit your hypotheses. Why was food something you considered, even if it ended up not significant?

**Answer**. *Thank you for your feedback. We initially developed a priori hypotheses that included psychological factors and food ingestion, considering them potential effect modifiers. While these factors did not significantly affect our results, we have described their non-significance in the results section. To address your suggestion, we have now included food factors in Figure 3 to provide a comprehensive overview of the variables considered in our study.*

**3.3. Table 2: I appreciate the addition of Table 2**, in which you appear to report all results, and not just the significant ones. Table 2 makes the paper much more cohesive.

**Answer**. *Thank you for your comment*.

**3.4. Table 2: 95% CI would be much more interpretable** to a reader than standard errors.

**Answer**. *Thank you for your suggestion. We have updated Table 2 to present the estimated coefficients with 95% confidence intervals (CI) instead of standard errors. This change should enhance the reader's interpretability. We maintained five of the seven models to maintain the cleanliness and simplicity of readability.*

**3.5. 200-202: this is a good added explanation** of how a reader can interpret your VIF.

**Answer**. *Thank you for your comment*.

**4. Discussion**

**4.1. Overall, the findings in the discussion section** seem consistent with the newly reported results in table 2.

**Answer**. *Thank you for your kind comment*.

**4.2. 238-263: as mentioned in the last round**, these sources should be part of your introduction to help motivate the study, since they would presumably inform the hypotheses that you’re testing.

**Answer**. *In response to your suggestion, we have revised the introduction to include the sources mentioned. These sources help better motivate the study and provide a clearer context for the hypotheses being tested.*

**4.3. 259: meta-analysis should not be capitalized**.

**Answer**. *We made your suggestion and changed it to lowercase letters*.

**4.4. 265: your study is on sex differences**, not gender differences.

**Answer**. *We changed the word sex instead of gender, and it is fine in the context. Thank you*.
