# Supplementary material for: Influence of adiposity and sex on SARS-CoV-2 antibody response in vaccinated university students: A cross-sectional ESFUERSO study
Source: PLOS Glob Public Health. 2024 Jul 26;4(7):e0002686. doi: 10.1371/journal.pgph.0002686 (PMC11280215; doi:10.1371/journal.pgph.0002686)
Supplement: Attachment — Submitted filename: Peer review comments from Mateus_Art5_R3.docx [file pgph.0002686.s007.docx]

**Peer review comments from M**

**Title:**

Influence of Adiposity and Sex on SARS-CoV-2 Antibody Response in Vaccinated

University Students: A Cross-Sectional ESFUERSO study.

**Abstract**

OK

**Introduction**

OK

**Methods**

Lines 113-116: A total of 116 students contacted but 108 gave blood samples. What happened to the remaining 8 students? Did they decline to give consent for study? Please clarify.

Is it the same number (108) that attended the survey questionnaire? Or 116? If the study targeted 500 participants and only 108 were included, did the authors check if this figure is sufficient to allow them extrapolate their findings?

- I did not find answers to the above comments. Maybe I am not able to understand it. So, I leave it to other reviewers.

**Ethics**

Line 143-148: Please add more details here on the fact that participants got sufficient explanation about benefits and risks for the study and they were free to withdraw from the study any time.

- I did not see this in the manuscript. Maybe it is not relevant. So, I leave it to other reviewers.

**Results**

I have difficult to understand the way results are presented. So, I leave this to another reviewer.

**Discussion**

OK

**Conclusion**

Line 276: Can you write conclusion as a title as you did for Introduction, Methods, Results, and Discussion?

- This was not addressed. Maybe it is not relevant. So, I leave it other reviewers.
