# Supplementary material for: Influence of adiposity and sex on SARS-CoV-2 antibody response in vaccinated university students: A cross-sectional ESFUERSO study
Source: PLOS Glob Public Health. 2024 Jul 26;4(7):e0002686. doi: 10.1371/journal.pgph.0002686 (PMC11280215; doi:10.1371/journal.pgph.0002686)
Supplement: Attachment — Submitted filename: Answer to Reviewer comments v09.docx [file pgph.0002686.s004.docx]

Dear Editors and Reviewers:

Thanks for the time you spent reviewing our paper. The reviewer comments enhance the quality of our paper title: Adiposity and Sex Influence on SARS-CoV-2 Antibody Response in University Students—an ESFUERSO cross-sectional study.

1. **Reviewer #1**: The study explored an important area of significant public health interest. It adds important findings to the already existing information and opens a window for more research. The methodology is explicit and supported by rigorous statistical analysis. Ethical issues have been taken in to consideration and the language used is explicit. The main findings are well presented. Limitations of the study have been highlighted for appropriate interpretation of results.

**Answer**. *Thanks for your kind comments*.

2. **Reviewer #2**: Manuscript is good, clear and recommend to publish with minor revision. First of all, there is need to describe more preciously figure 1 and figure 2, or present those figures in tables.

All other section are clear.

**Answer**. *Thank you for your constructive comments. We have revised the figure legends to provide more detailed descriptions:*

*Figure 1: Sex Differences in Neutralizing Anti-RBD IgG Ratios as a Function of Body Fat Percentage. The revised legend for Figure 1 now details the relationship between neutralizing anti-RBD IgG ratios and body fat percentage among university students. It utilizes the Lowess smoothing technique to delineate trends, with separate markers for females (diamonds) and males (crosses). The trend lines are differentiated by a continuous line for females and a dashed line for males, visually emphasizing the sex differences in the data.*

*Figure 2: Polynomial Regression Analysis of Anti-RBD IgG Ratios Predicted by Body Fat Percentage. The legend now describes the polynomial regression applied to illustrate the relationship between anti-RBD IgG ratios and body fat percentage, highlighting the varying responses between sexes. Females are represented with white circles and fitted with a third-degree polynomial, indicating a more complex response pattern. At the same time, males are shown with black circles and a second-degree polynomial, reflecting a more straightforward response curve.*

*Thanks for your comments, which have enhanced the clarity of the figures and their legends. We are open to further suggestions to improve our manuscript.*

3. **Reviewer #3**: Thank you for your comments. In brief: “I think the paper is potentially an important contribution to the field but requires additional writing particularly in the background and methods sections, and at least one additional table.”(sic by reviewer).

3.1. “The background section is a little sparse. At the moment, I’m a little unclear as to how this study was motivated. It’s also not clear what your main independent variable was before you ran the analysis. Was it the SISCO measures? Or was this an exploratory study to see what factors account for immune response? If so, it would be helpful to state that.”

**Answer**. *Thank you for your feedback. We expanded the background section to explain the study's motivation comprehensively. The motivation for our research stemmed from our previously published work investigating sex-based differences in metabolic outcomes. Building on this foundation, our current study examines how these factors—specifically sex and body fat percentage—affect the immune response to COVID-19 vaccination.*

*The current investigation continues our efforts to understand how various biological factors, such as adiposity, contribute to individual variations in immune response to vaccination.*

*By providing this context, we aim to clarify that our study was not merely exploratory but driven by specific hypotheses based on our earlier research. We appreciate your suggestion and believe that these amendments will make the motivation and direction of our study more transparent to the readers.*

*The SISCO questionnaire assessed distress, uncertainty, lack of sleep, sadness, and anxiety. We used the original name to avoid confusion with other questionnaires. It was used in Mexico to evaluate students during the pandemic.*

3.2. “How and why did you choose the factors to analyze? You’ve provided some justification for assigned sex at birth and adiposity in the background, but not for other covariates or for your main outcome. Though it’s not clear to me that you’re actually building models for sex and adiposity that would adjust for the right confounders. Does the literature point the way towards any covariates that would be important to include in measuring the effects of adiposity and sex on immune response? Is there literature on other vaccines and interactions between adiposity and sex?”

**Answer**. *Thank you for raising these critical points. Our choice of factors for analysis was guided by a review of the medical literature, which has documented variations in immune responses based on sex. Furthermore, our prior research has identified sex-specific biochemical differences in metabolism, which we believe could also influence immune response.*

*In the context of COVID-19, while one study cited in our manuscript has explored the relationship between BMI and immune response, our analyses indicated that body fat percentage provided a more precise measurement with less error. Therefore, we focused on body fat as a more accurate representation of adiposity.*

*To address potential confounders, we carefully considered the existing literature on vaccine response, which suggests that factors such as age and underlying metabolic conditions (i.e., including family history of hypertension and diabetes) might impact immune response. Thus, we adjusted for these covariates in our models to isolate the effects of sex and adiposity more accurately. We also considered insights from studies on other vaccines where interactions between adiposity and sex have been reported to ensure our models were comprehensive.*

*We have provided additional references and a more detailed explanation in the manuscript to justify our choice of covariates and to elucidate our analytical approach. These revisions will clarify the rationale behind our methodology.*

3.3. “Much more information is needed in the methods section on how all of your measures were operationalized. E.g. you note a “metabolic risk group” in 173-174, but didn’t describe in methods how you created this group. How was vaccination assessed? Was it self-report, administrative records, medical records, something else? How were body fat percentage, weight circumference, neck circumference, and blood pressure assessed? It’s important to describe for all those measures, but particularly for body fat percentage as it’s central to your analysis.”

**Answer**. *We included more information on the methods, including a reference to previous papers. Thank you for highlighting this point.*

3.4. “Every model that you ran should be described in the methods section, and ideally there should be a table with your results (including the non-significant ones). The way I understand it, you ran a separate model for each of the 6 factors listed at the bottom of Table 1 plus another for food consumption, though that number may be a lot more since it appears as if you tested different models for interactions separately. Or was it just one model with all of the factors loaded in? Describing it in the methods section would greatly clarify how many models for the reader, which is important because depending on how many models you ran, there starts to become an increased risk that that many tests of significance may result in something statistically significant just due to the sheer volume of tests.”

“It’s still not clear to me which model you ran includes the interaction term for fat percentage and sex.”

**Answer**. *Thank you for your meaningful feedback. We understand the importance of transparency in reporting our analytical approach and the potential implications of multiple testing. In the revised methods section, we have now provided a detailed description of each statistical model run, including the rationale behind including each variable and interaction term.*

*Specifically, we ran a separate model that included the first-order interaction term with sex, as we hypothesized that this interaction might be significant based on our previous publications. These interactions were not exploratory but were included based on priori hypotheses derived from our past research.*

*We ran seven models, each including an interaction term for body fat percentage and sex, which we explicitly indicated in the methods section. To further clarify our analysis and address concerns about the risk of false positives, we have included a comprehensive table of results in the manuscript, which presents the outcomes for all models, both significant and non-significant. This transparency ensures that readers can fully evaluate the extent and impact of our testing.*

*By limiting our models to clinically meaningful variables and interactions and reporting these in the manuscript, we aim to support our statistical findings. We hope this approach and the additional information provided helped to clarify our methods and the results obtained.*

3.5. “Table 1: I see food consumption as something that was measured and analyzed (195). How that was measured and operationalized should be described in methods, and should be included in table 1.”

**Answer**. *Food consumption did not show any effect or association with our main objectives. As this issue distracted our readers from the study's aims, we eliminated the sentence.*

3.7. “The dataset was not available at the provided link (it may have required a UT:RGV login)”

**Answer**. *The dataset is included in the submitted document. The data will be publicly available when the publication process is complete.*

3.8. “There’s not enough information in methods currently to assess whether the results back up the discussion points.”

**Answer**. *We added information on the methods used.*

3.9. “216-248 is well-written and highly relevant. These studies should have been mentioned in the background to establish the literature and motivate the current study.”

**Answer**. *Thank you for acknowledging the relevance of sections 216-248 and for your constructive suggestion, which enriches the background of our study.*

*We highlighted key findings from previous studies (others and ours) that have explored the relationships between sex-specific factors, metabolic profiles, and immune responses.*

*The added background information contextualizes our study within the broader field of research, emphasizing its novelty and the potential implications of our findings for personalized vaccination strategies and public health policies.*

Line-by-line comments:

3.10. “113-114: Do you mean the parents of the students? Or the initial cohort?”

**Answer**. *We corrected the text. Thank you.*

3.11. “114: Why were the students contacted due to the pandemic? Why was it this subset in particular (random, administrative reasons, etc.?) and not the entire cohort?”

**Answer**. *We corrected the text. Thank you.*

3.12. “138: might be useful to include the distance and method of transport and/or summarize the sample preservation procedure during transport”

**Answer**. *We clarified the care we had for the samples. Thank you.*

3.13. “161/165: Is it 114 students or 108?”

**Answer**. *We corrected the text (n=108 students suitable for analysis). Thank you.*

3.14. “168-169: Unclear what you mean by “significantly” here, was it statistically significant? Or did they meet some pre-defined threshold?”

**Answer**. *Thanks for pointing out this ambiguity. We clarified the text: 70% of the students were identified as having T2D, hypertension, obesity, or a combination of the conditions. Thank you.*

3.15. “167: How was sex assignment determined? Was it self-report or administrative data?”

**Answer**. *Despite having previous information (administrative data), we asked direct questions using a questionnaire (self-report)*.

3.16. “184: Describe how to interpret this VIF for readers who may be unfamiliar with VIF.”

**Answer**. *We appreciate your suggestion and believe that this clarification enhances the accessibility and comprehension of our study's methodology.*

*In the Methods Section, we detailed the calculation of VIF, and in the Results Section, we elaborate on its interpretation in the context of our analysis. Specifically, the multicollinearity analysis revealed that the Body Mass Index (BMI) and body fat percentage presented a VIF of 4.8, translating to an inverse VIF (1/VIF) of approximately 0.02. This indicates a moderate level of collinearity between these variables.*

*To ensure clarity and maintain the regression coefficients' interpretability, we analyzed the impact of BMI and body fat percentage separately in our regression models. This approach allows us to disentangle the effects of these closely related but distinct aspects of body composition on the outcome of interest.*

*For the benefit of readers unfamiliar with VIF, we included a brief explanation: “**The multicollinearity analysis showed BMI and fat percentage had VIF= 4.8 and 1/VIF= 0.02. This suggested a moderate level of collinearity, so we analyzed separating both variables to maintain a clear interpretation of the regression coefficients.”*

3.17. “186: Given the limitations of this study, causality can’t be established here, so be cautious of using “explaining” to describe this relationship.”

**Answer**. *Thank you for this suggestion. The term “explaining” was used in the statistical context. We changed to “The body fat percentage interaction with sex was statistically significant, supporting the serum concentration of anti-RBD decreased as adiposity increased…”*

3.18. “Minor copyediting comments:

3.18.1. “58: unsure what “adeptly” is referring to here”

**Answer**. *Thanks for this observation. We corrected the mistake and eliminated the word.*

3.18.2. “100: Needs a citation”

**Answer**. *Thanks for this point. We added references on this point.*

3.18.3. “51: needs to be a sentence”

**Answer**. *We rewrote the sentence: “ This study focused on the Ig-G anti-RBD marker, analyzing its potential correlations with sex, vaccine type, body fat percentage, metabolic risk,...”*

3.18.4. “151-162: Could use some minor copyediting”

**Answer**. *We edited these sentences. Thank you for this suggestion.*

3.18.5. “167: “female students” rather than "females".

**Answer**. *We made your suggested correction*.

4. **Reviewer #4**: General comments:

4.1. “Well articulated molecular level understudy of vaccine, especially COVID-19 vaccines which was globally used in curbing the pandemic.

This study underscores multiple factors that could affect vaccine take, and can be extrapolated to other vaccines especially childhood RI vaccines looking at familial, environmental, dietary and other domains for further development of vaccines.

Studying younger age-groups as in this study helps to target larger percentage of the population pyramid and hence large vulnerable groups that are our future technocrats and leaders, therefore helping humanity in essence.”

**Answer**. *We appreciate your kind comments*.

4.2. Suggestions:

4.2.1. “Title: Adiposity and sex influence in vaccinated university students on SARS COV-2 Antibody response: An ESFUERSO cross- sectional study.”

**Answer**. *Thank you for the suggestion. We modified the title to Influence of Adiposity and Sex on SARS-CoV-2 Antibody Response in Vaccinated University Students: A Cross-Sectional ESFUERSO study.*

4.2.2. Key words: COVID-19 vaccines; sex; adiposity; immune response; regression analysis; anti-RBD

**Answer**. *Thank you for this suggestion. We modified the Key words: COVID-19 vaccines; sex; adiposity; immune response; polynomial model; regression analysis; anti-RBD.*

4.2.3. “Methods: Line 137-these aliquots were subsequently”.

**Answer**. *We corrected the text. Thank you.*

4.2.4. “Statistical analysis: Line 151- with percentages for count variables”

**Answer**. *We corrected the text. Thank you.*

4.2.5. “Results: Line 169-conditions were identified”

**Answer**. *We corrected the text. Thank you.*

4.2.6. “Line171- only 3(3%) of the students”

**Answer**. *We corrected the text. Thank you.*

4.2.7. “Line 189- the interaction remained in spite of the vaccine types.”

**Answer**. *We corrected the text. Thank you.*

4.3. “References: Other COVID-19 vaccine studies in younger age groups globally could be relevant?.....add a few please”

**Answer**. *We added more references distributed in the text. Thank you.*

**5. Reviewer #5**.

**General comments:**

This is a very good study that add value to the literature. However, it need improved by considering the above comments before moving to publication.

**Answer***. Thank you for the comments on our paper. We divided your comments and our answers into two separate sections: concepts and format suggestions.*

*Our primary intention was to give a concise paper that tested sex as an essential variable to define metabolism and immune response based on our previous publications. Thank you for your advice. We understand the manuscript was so concise that you require justified clarification on some issues. We added most of your suggestions (even though we modified the paper's title). Still, we tried not to increase the wording volume simultaneously, especially with non-significant results.*

5.1. Conceptual comments:

5.1.1. “Abstract. Line 66: “… this trend was …” => which trend?”

**Answer**. *The text from line 65 to line 67 was: “In males, increased adiposity was associated with a decrease in Ig-G anti-RBD concentration, while in females, the response increased. Importantly, this trend was consistent regardless of the vaccine received.” We modified it to: “In males, increased adiposity was associated with a decrease in Ig-G anti-RBD concentration, while in females, the response increased. Importantly, this pattern remained consistent regardless of the vaccine received.”*

**Introduction**

Lines 92-99: “This group of students reported … psychological stress”. This paragraph looks like results rather than introduction. Can this be clarified?

**Answer**. *We decided to eliminate in the Introduction section some of the characteristics we found in our study and reduced the wording to: “The present study focused on a nested sample of students from the ESFUERSO program, which recruited first-year college students living in Reynosa before the COVID-19 pandemic in 2018. These students experienced the impact of the COVID-19 pandemic, forcing them to home confinement, which led to changes in their habits.”*

Lines 99-100: “Vaccination 99 efforts began in 2021, employing novel mRNA vaccine used in veterinary science for three decades”. => Did authors do vaccination as part of this study (method)? If not, then this sentence needs reference if it comes from the literature.

**Answer**. *Thanks for this observation. We added a reference to clarify that it is coming from scientific literature [34522017].*

**Comment for the introduction**: This section of the manuscript is too short, less than one page is not sufficient. There is no clear rational of the study described here. Authors need to screen further literature to make this section rich and explain why this study is important and/or needed.

**Answer**. *This is an important point. We studied the relationship between sex and obesity's effects on different metabolic conditions. We had the opportunity to understand the effect of both on immune response. Considering some suggestions from this review, we added the importance of the sex interaction with adiposity. [Fischinger PMID: 30547182] and some of our studies [Garcia-Oropesa PMID: 37660428], [Garces-Hernandez PMID: 36286282], [Villanueva-Ortega PMID: 31151875], Females showed physical activity effects on immunological markers [Landeros PMID: 25091615], in other studies we controlled by sex to blunt metabolic differences attributed to sex [Garibay-Nieto PMID: 34890785], migration effects on adiposity in obese women [Gonzalez Barranco PMID: 11421108].*

*We added the following sentences: Our previous studies have demonstrated sex's influence on children's metabolism in different contexts [Garibay-Nieto PMID: 34890785, Garces-Hernandez PMID: 36286282], [Villanueva-Ortega PMID: 31151875]. Physical activity and migration have shown an influence on immunological markers and health outcomes [Gonzalez Barranco PMID: 11421108], [Landeros PMID: 25091615].*

**Methods**

Line 111: Better to avoid confusion: is the ESFUERSO study the one that is being published in this manuscript or different? How sample size was calculated? 500? 116? 108? Better to stick on sample size for your manuscript and explain how you got this figure. Did the authors check the power for this sample size to be representative of the studied population? A subset of 116, was it for what (survey, blood collection or both)?

**Answer**. *Thank you for this important point. We clarified the text and provided more details on the methods.* *The ESFUERSO study (Estudio en la Frontera Urbana para las Enfermedades y factores asociados a la Obesidad) aimed to assess factors associated with obesity among university students. Detailed methodology is available in Oropesa et al. (PMID: 37660428). In brief, the study targeted 500 first-year students from Universidad Mexico-Americana del Norte (UMAN) and Universidad Autonoma de Tamaulipas (UAT), who provided informed consent and completed validated questionnaires on family metabolic risk derived from three previous pilot studies. These questionnaires, demonstrating reliability with Cronbach’s alpha ranging from 0.72 to 0.96 and kappa coefficients for categorical variables between 0.5–0.91, were designed to capture differences by sex, physical activity, and dietary habits, particularly among students outside Reynosa.*

*For the present study, a nested cohort during the COVID pandemic and vaccines accessible were invited to answer a questionnaire and sample drawn at the University Campus to ensure representativeness and minimize bias, with anthropometric and clinical measures including weight, height, waist circumference, and blood pressure taken under standardized conditions. Body Mass Index (BMI) was calculated using WHO criteria. Measurements were conducted by ISAK-certified dietitian-nutritionists using established techniques and bioelectrical impedance (Tanita TBF-300A) for body fat percentage. Blood samples collected after an overnight fast were analyzed for biochemical parameters such as liver enzymes, lipid profile, insulin, glucose, and creatinine.*

Line 110: Based on the content of this paragraph, this title should be: Study sample, design, and data collection. Please describe the study design for your research (it seems to be a quantitative clinical study and comparative, I guess). Did the authors respect GCP procedures? Were there SOP and training of study staff done? What was done for the safety of study participants and staff? Explain in details how survey was conducted and how participants were selected (sampling method). Explain in details what anthropometric measurements were done. What did authors do with blood samples collected? What lab facility was used for analysis, for what kind of results?

**Answer**. *Thank you for your feedback. We included a more detailed description of our study's sample, design, and data collection processes. Our study is an observational study design aimed at evaluating the antibody response after COVID-19 vaccination, considering the effect of sex and body fat percentage. The participants are part of a nested sample coming from the ESFUERSO study, in the third year of follow-up. We added a recently published paper with detailed methods used in this cohort.*

*Good Clinical Practice (GCP) and Training: The research team adhered to Good Clinical Practice (GCP) guidelines throughout the study to ensure the integrity of the data collected and the safety of all participants. Standard Operating Procedures (SOPs) were developed and strictly followed. Prior to data collection, all study staff underwent comprehensive training on these SOPs, including ethical conduct, data collection techniques, and emergency response protocols.*

*Safety Measures: To safeguard the health and well-being of participants and staff, especially considering the COVID-19 pandemic, we implemented a COVID-19 protocol based on guidelines from health authorities and our universities. This included regular health screenings, the use of personal protective equipment, social distancing measures, and sanitation protocols in all study environments.*

*The survey was conducted using electronic devices, but in school rooms and with access to personnel in case participants have doubts. Those students able to respond to our call were included.*

*Each participant's anthropometric measurements, including height, weight, body mass index (BMI), and waist circumference, were taken following standardized procedures to ensure accuracy and reliability.*

*Blood samples were collected at the university's dedicated facilities, adhering to strict safety and hygiene standards. Samples were analyzed at the specialized and standardized Central Laboratory in Hospital General de Mexico and the Biomedical Institute at UNAM. The samples were transferred in cold containers with dry ice. We appreciate the opportunity to clarify these aspects of our research.*

Line 127: “… the Coping Inventory for Stressful Situations (SISCO),…” => is it CISCO or CISS? Please check. It seems that the system used was CISS, not CISCO. Is there any relationship between stress and adiposity? It is not seen in the background.

If you are trying to link adiposity and immunogenicity, how the CISS components fit into the analysis here? Are you comparing the level of immunity of people who are affected by stress to those with adiposity? Please clarify. CISS components can be cofounding factors, how do you deal with that? There is possibility of having 3 groups of participants to compare: those with stress only, those with adiposity only, and those with both stress and adiposity. Please specify what you are trying to do for this study. It is still not clear.

**Answer**. *Thank you for pointing out the confusion regarding the Coping Inventory for Stressful Situations. To clarify, we used the Spanish version of the instrument, which is indeed named SISCO (Modelo SIStémico COgnoscitivista para el estudio del estrés académico), not related with the CISS. We apologize for any confusion and have corrected the acronym to SISCO throughout the manuscript to reflect this accurately.*

*In our study background, we explore the relationship between stress and adiposity, which, while not extensively detailed, serves as a foundational element for integrating the analysis of stress (measured by SISCO) with adiposity and its potential impact on immunogenicity. Emerging research suggests that psychological stress can influence the body's fat distribution and immune response, thereby providing a rationale for examining these factors together.*

*Psychological stress, as measured by SISCO, alongside adiposity, may interact to influence the immune response. We isolated their individual and combined effects on immunogenicity by including stress levels and adiposity measures as covariates.*

*These clarifications enhance the manuscript's coherence and contribute to a better understanding of our findings.*

Lines: 130-142: Why this section is under Measures? It is better to have another sub-heading named “Data collection” and put this information there. The Measures could contain only information about weight and height and how it was done. Were the materials used calibrated and quality checked? If the survey was administered electronically, what if participants needed clarifications for some questions before responding? What was put in place to solve that and avoid bias in the responses provided?

**Answer**. *We included more information on the methods. Thank you for this suggestion.*

Lines 134-136: **“Blood samples were obtained after an overnight fasting period for the measurement of serum concentration of Ig-G anti-RBD by indirect ELISA”** => What was the real time set for blood samples collection to avoid participants being kept fasting for long time? Was there payment for time and transport compensation? As the survey was online, how participants who gave blood get in contact with study staff to provide their blood? Was there a dedicated study site where they could go or staff could move to their home to collect blood?

Lines 144-148: Was the consent voluntary or paid? Was there any study information sheet given to participants? Did they get explained the study procedure before accepting to take part of it? Were they given opportunity to withdraw from the study any time when they feel to do so?

**Answer**. *We are grateful for asking for safety measurements for our study participants. In response to your inquiries.*

*Blood samples were collected in the morning to minimize the fasting duration (samples were drawn between 7 am to 9 am) and standardize the conditions for serum analysis.*

*While our study did not offer economic or academic compensation, we ensured that participants were fully informed about the requirements and that their participation was voluntary.*

*Participants willing to participate signed an informed consent and initially engaged with the study through an online survey. Upon completion, they provided blood samples at the study site located at the university laboratories.*

*The informed consent, approved by the Institutional Review Board (IRB), explicitly stated that participation was entirely voluntary and that participants could withdraw at any point without any repercussions.*

*We have briefly mentioned these logistics in our manuscript and provided a reference to a newly published paper detailing the methods we employed. For example, the Blood samples were collected in the morning, within a narrow window from 7:00 AM to 9:00 AM, to minimize the fasting duration and standardize the conditions for serum analysis.*

Also specify what are the inclusion/exclusion criteria for participants. Did you include people who are only vaccinated (fully, partially, or not vaccinated)? How did you check the vaccination status? Or did you vaccinate them yourself as a prospective study? How did these criteria apply for survey and blood collection?

**Answer**. *Here are the details regarding how eligibility criteria were applied:*

*Our study included adults aged 18 and above who were eligible for vaccination per the national vaccination guidelines at the time of the survey.*

*We included individuals who had received their first doses of the vaccine and those who were not vaccinated at the time of their participation in the study. The participants’ vaccination status was verified through self-report and physical evidence (vaccine name).*

*The study did not involve administering vaccinations; instead, we assessed the immunogenic response among participants based on the vaccination campaign conducted by the government in their clinics.*

What kind of analysis did you perform (univariate, bivariate or both)?

**Answer**. *Thank you for the opportunity to clarify the statistical analysis conducted in our study. The analysis proceeded as follows:*

*We conducted descriptive univariate analyses to understand the basic distribution and characteristics of the variables.*

*We then moved to bivariate analyses, specifically employing locally weighted scatterplot smoothing (LOWESS) regressions. This approach allowed us to visually explore and capture the best mathematical function describing the variation in anti-RBD concentration associated with two key variables of interest: sex and body fat percentage.*

*The LOWESS regressions revealed an apparent interaction effect between sex and body fat on anti-RBD concentration. This finding was consistent with our hypothesis, grounded in previous research we published on metabolic differences between sexes and their potential impact on immune response. We further explored these interactions through multiple regression models, incorporating the combined effects of sex and body fat on anti-RBD concentration.*

*.*

**Results**

Line 165: “… 108 students …” => Be consistent, what is the real sample size for this study?

Lines 165-176: We do not see the data here. There is interpretation of data that are not presented here. Please present your data before making any interpretation or comment.

**Answer**. *Thank you for highlighting these essential points for clarification.*

*The reference to "108 students" pertains to a specific subset of participants from a larger cohort study that initially involved 500 students. This larger cohort was established before the pandemic to monitor various health parameters. As the pandemic started, we sought to investigate the impact of COVID-19 on immunogenic responses among these students, leading to a follow-up study.*

*We included the students based on their accessibility and willingness to participate in the study. We revised the manuscript to include a detailed presentation of the data collected.*

Line 176: What do authors mean by “positive anti-RBD”?

**Answer**. *Thank you for the opportunity to clarify what we mean by “positive anti-RBD” in our study. In the context of our research, a “positive anti-RBD” result refers to a serum sample with an antibody concentration exceeding a predefined threshold, as determined by the enzyme-linked immunosorbent assay (ELISA) method. This threshold is critical for distinguishing between individuals who have likely been exposed to the virus and developed an immune response, as evidenced by the presence of antibodies against the receptor-binding domain (RBD) and those who have not. Using a quantitative approach enables us to gauge the concentration of these antibodies.*

The way table 1 is presented, it looks like sex is the dependent variables that you are trying to link to all other variables in the first column. It could be better to have sex in the same column with the other variable (column 1) and put data about immunogenicity at the place of sex. Did you have sub categories for the independent variables (e.g. for age: young, medium adult and older)? Was the immunity the same for all of them?

**Answer**. *Thank you for your valuable feedback on the presentation of Table 1. In our study, the primary variables of interest were the interaction between sex and body fat percentage and how these variables influence antibody concentration. Sex, a categorical variable, was central to our analysis as a crucial factor in understanding the distribution of other variables, including immunogenic response.*

*The variable sex in the whole manuscript is presented as an independent variable rather than misconstrued as a dependent variable. The dependent variable in our analysis, antibody concentration (indicative of immunogenicity), is continuous and addressed in the regression analysis as a dependent variable.*

*Finally, the reviewer should consider maintaining statistical efficiency and study design. We did not contrast a wide range of ages (this is a young population). We analyzed the variables we considered could be essential for immunity, and we were able to collect them.*

Lines 184-196: This seems to be a bivariate analysis linking sex and adiposity. This should be clearly specified in the method. Why comparison between males and females only? You should do the same for other variables too. You should also compare fat and non-fat people, people with hypertension and those with normal blood pression, older and young, those with metabolic risk factors with those without risk factors, etc.

**Answer**. *Thank you for your feedback regarding the scope of our analysis and the clarity of our methodological approach. Our study primarily aimed to explore the association between sex (males and females) and body fat percentage, as this was directly aligned with our research objectives and hypotheses concerning the potential differential immunogenic responses between genders about adiposity.*

*Our study group's unique composition is university students with a relatively narrow age range (mean age 22.6, SD 1.3 years). This age homogeneity reduces the variability and potential confounding impact of age on our primary analysis. Preliminary evidence suggests that sex-specific metabolic and immunological differences could significantly influence the body's response to vaccination, making the sex-adiposity interaction a compelling area for investigation.*

*We added a new table with a detailed description of the model's construction, considering the measured dimensions of the variables. These enhancements address your concerns and enrich our study's findings and implications.*

Lines 194-196: This is an interpretation of data that are not presented in the manuscript. Can you present a figure here before making interpretation?

You have only 2 figures, all other data are not presented but have interpretation and comment.

**Answer**. *Thank you for highlighting the importance of clearly presenting data to support our interpretations.*

*In addition to referencing Table 2, which provides a detailed description of the statistical models used and the outcomes of these analyses, we stated in the text those variables with no statistical significance.*

*Furthermore, we have reviewed the manuscript to ensure that all interpretations and comments are supported by either data presented in tables or figures, enhancing the transparency of our findings. We believe these revisions and additions address your concern and improve our study's results' overall presentation and robustness.*

Were males and females receiving the same type of vaccine? This should be clarified. If they received different type of vaccine, it may be difficult to compare these findings!

**Answer**. *Thank you for raising an important point regarding the potential impact of vaccine type on our findings. Our analysis showed no significant differences in vaccine type distribution between male and female participants. This information is detailed in the Results section of our manuscript, where we report that the vast majority (97%) of the student participants received either the Moderna or Pfizer vaccine, with no statistically significant difference observed between sexes in terms of which vaccine they received.*

*These findings suggest that the type of vaccine administered did not vary systematically by sex in our study cohort, allowing us to make comparable analyses between male and female participants regarding the outcomes of interest.*

**Discussion**

Lines 214-215: “Stress scores do not appear to have significant effects” => I did not see data in your results showing this.

**Answer**. *Thank you for pointing out the need for a more straightforward presentation of the data concerning stress scores and their effects. To address this, we have introduced a new Table 2 in the revised manuscript, which provides a detailed description of the statistical models analyzed, including the effects of stress scores as measured by the SISCO inventory. The stress scores did not significantly correlate with the antibody concentration outcomes we measured.*

Lines 216-219: The introductory sentence in this paragraph gives link between sex and immune response but the example given link sex and adiposity. It is better to give the right example here.

**Answer**. *We have revised the paragraph; in steps, we described the reported effect on sex and other conditions in Mexico that affect the immune response. Yamamoto addressed sex and adiposity, which we describe in the manuscript in a paragraph below.*

*By doing so, we aim to clarify the narrative and ensure that the example given accurately illustrates the complex interplay between sex, adiposity, and immune response.*

Lines 230-232: “This study highlighted the heightened vulnerability of individuals with underlying health conditions or obesity to SARS-CoV-2 infection” => I did not see data in the analysis supporting this statement.

**Answer**. *We addressed the Montes-Herrera study, not ours. To avoid confusion, our revised statement now reads: “**The authors emphasized the vulnerability of individuals with underlying health conditions, including obesity, to SARS-CoV-2 infection, a concern that is particularly pronounced in older populations, as demonstrated by studies conducted in Mexico. While our study focuses on a younger cohort, it underscores the importance of considering such vulnerabilities across diverse age groups.”*

*Thanks for this advice.*

Line 235: “Other populations have reported similar findings …” => what authors mean by other population? Does it mean study conducted out of Mexico? It should be clear.

**Answer**. *The other population was the Yamamoto paper, and we stated that the study was conducted in Japan.*

5.2. Format comments:

*Dear reviewer. We addressed each of your comments and modified the text as suggested. Thank you so much for the enrichment.*

**Introduction.** Line 76: Insert references after the word conducted.

**Answer**. *We added the references. [Zhangkai Jason Cheng, PMDI: 34451994, Kodde PMDI:36851326, Su Youn Nam, doi:10.1001/jamanetworkopen.2022.12996]*

Line 80: Why reference comes after a dot (.)? => .(1) should read (1). Please correct this in the whole document.

**Answer**. *The document was corrected.*

Line 87: “BMI > 40 kg/m2” should read “BMI > 40 kg/m^2^”.

**Answer**. *The correction was made.*

Line 90: If ESFUERSO is an abbreviation, can it be explained in full here?

Th ESFUERSO, is acronyms by Estudio de la Frontera Urbana para las EnfeRmedades y factores aSociados a la Obesidad

Line 88: Insert reference after the word responses.

Lines 91-92: “… which recruited first-year college students living in Reynosa prior the

COVID-19 pandemic in 2018”. => this sentence should be part of methods section, not introduction.

**Answer***. The correction was made and the phrase is deleted.*

Lines 102-103: “The aim of this study was …” => start this as a new paragraph.

**Answer**. *The correction was made.*

Lines 105-106: “The study provides insights in the immune response and the potential implications in the context of COVID-19”. => This sentence should come later in the results or discussion.

**Answer***. The correction was made, and the phrase was deleted*.

**Methods.** Lines 151-152: “Descriptive statistics with percentage for counts variables, mean and standard deviation for continuous variables”. => This is not a sentence, please correct grammar issue here.

**Answer**. *The Correction was made. Descriptive statistics included percentages for categorical variables, means with standard deviations or medians, and interquartile range (Q1, Q3) for continuous.*

Line 152: CISCO or CISS?

**Answer**. *The name of the questionnaire is SISCO. We clarified the name.*

Line 158: “… lineal models …” or “linear models”?

**Answer**. *The correction was made*.

Line 161: “… with 114 students with all complete data”. => What is the real sample size for this study (500, 116, 108, 114)?

**Answer**. *We clarified this issue. The analyzed sample size for this study was 108.*

Add a title of “Data presentation” under which you explain how your data are presented in this manuscript: graphs (bar chart, trend lines, pie chart, …), table, text, etc.

**Answer**. *We clarified the use for each type of data presentation, referring to the corresponding data presentation.*

**Results.** Line 177: In the table “BMI (kg/m^2)” should read “BMI (Kg/m^2^)

**Answer***. The correction was made.*

Line 177: Data in table 1 are confusing to me. Please explain what is in brackets, dot (.) for some and comma (,) for others.

**Answer**. *The table has been revised. Continuous variables are displayed as mean and standard deviation, while ordinal variables are presented as median and interquartile range (Q1, Q3).*

Line 188: Ab-RBD: What this abbreviation means? Better to be consistent in the whole document.

**Answer**. *The correction was made*.

**Discussions.** Line 229: Insert reference after the word “index (BMI)”.

**Answer**. *We added the reference (PMID: 37627889).*

Line 242: “… this outcomes” should read “these outcomes”.

**Answer**. *The correction was made.*

**Note:** I realise that there is no conclusion in this manuscript. Please add this section. Lines 243-248 should be part of the conclusion. Authors may add other important information such open window for new research. It is important to have research looking at COVID-19 vaccine protection level in a population with other weight for example.

**Conclusion**

To be added, see comment above.

**Answer**. *We used the word “In summary”; now it is “In conclusion”.*
